# Combined Effect of Nitrogen Fertilizer Application and High Temperature on Grain Quality Properties of Cooked Rice

**DOI:** 10.3389/fpls.2022.874033

**Published:** 2022-04-18

**Authors:** Yanqiu Xu, Xianyue Guan, Zhanyu Han, Lujian Zhou, Yan Zhang, Muhammad A. U. Asad, Zhaowen Wang, Rong Jin, Gang Pan, Fangmin Cheng

**Affiliations:** ^1^Department of Agronomy, College of Agriculture and Biotechnology, Zhejiang University, Hangzhou, China; ^2^Jiangsu Collaborative Innovation Center for Modern Crop Production, Nanjing Agricultural University, Nanjing, China

**Keywords:** rice (*Oryza sativa* L.), grain quality, high temperature, nitrogen fertilizer, interactive-effect

## Abstract

Ambient temperature and nitrogen (N) fertilizer are two of the most important factors that affect rice grain quality. However, less information has been available on the interactive effect of N fertilizer and ambient temperature on grain quality under stressful high temperature (HT). In this article, the effects of panicle N fertilizer, ambient temperature, and their interaction on starch composition, particle size distribution of starch granules, starch physicochemical properties, and storage protein accumulation in milled grains were investigated to clarify the potential role of panicle N fertilizer topdressing in regulating rice grain quality under stressful HT by using a two-factor experiment of three N levels in combination with two temperature regimes. Results showed that appropriate application of panicle N fertilizer could attenuate the adverse effect of HT during grain filling on milling quality and chalky occurrence to some extent, particularly for the effective alleviation of HT-induced decrease in milling quality. However, the topdressing of panicle N fertilizer tended to enhance starch gelatinization enthalpy (ΔH) and its setback viscosity in HT-ripening grains, with the simultaneous decrements in the number and surface area proportions of smaller starch granules under the higher N fertilizer in combination with HT exposure. The effects of higher nitrogen fertilizer and HT exposure on total protein content and gluten composition of grains were additively increased. Hence, the topdressing of panicle N fertilizer exacerbated HT-induced deterioration in cooking and eating quality, rather than alleviating the negative impact of HT exposure on the palatability of cooked rice.

## Introduction

Rice (*Oryza sativa* L.) is the staple food for more than half of the world population. In the last half-century, rice yield was dramatically increased due mainly to the improvement in breeding and cultivation technologies (Peng et al., 2008; Bao, 2012). With the improvement of living standards, grain quality has become increasingly concerned by many rice consumers (Fitzgerald et al., 2009). On the other hand, the rice cultivars with high yield often had relatively poor grain quality (Bao, 2012; Gu et al., 2015). A well-known example is that most of the newly released “supper rice” cultivars in China were typically characterized by more spikelets per panicle, thereby leading to much higher yield potential per hectare as compared to the conventional cultivars (Zhu et al., 2017; Wei H. H. et al., 2018). However, the relatively poor grain quality and inferior palatability of “supper rice” are generally contradictive with the farmers’ preference to the rice cultivars with desirable quality, which is one of the important constraints for the economic benefits of “supper rice” cultivars in rice production (Zhu et al., 2017; Zhou et al., 2020). Rice cultivars with both high yield and superior grain quality are still rare. Furthermore, some traditional measurements of agronomic practices to achieve higher rice yield were also reported to have a negative impact on grain quality (Fitzgerald et al., 2009; Ning et al., 2020). Thus, it is urgent to synergistically increase rice yield and improve grain quality through genetic improvement and proper agronomic practices.

Rice grain quality is assessed by appearance, milling, cooking, and eating, and nutritional traits, which are closely associated with various aspects of the physical property and biochemical composition of rice grains as well as their milling processes (Fitzgerald et al., 2009). Starch is one of the main components in rice grain, so that its physicochemical parameters such as apparent amylose content (AAC), gelatinization temperature (GT), gel consistency (GC), and rapid visco-analyzer (RVA) pasting viscosity have been set up to evaluate the cooking and eating quality (Chrastil, 1992). It has been well documented that grain quality is dependent on both environment and genotype (Bao, 2012). Among the environmental effects on grain quality, climatic conditions and agronomic management are the important factors that influence rice grain quality (Fitzgerald et al., 2009; Patindol et al., 2015). High temperature (HT) evidently shortened grain-filling duration and reduced grain starch accumulation, consequently leading to the markedly increased chalky fraction and chalky area due to insufficient grain filling (Dou et al., 2017; Cheng et al., 2019). The deterioration of grain quality for rice plants being exposed to HT was also ascribed to the inferior palatability of cooked rice by affecting grain starch physicochemical properties (Geigenberger, 2011; Jing et al., 2021). Furthermore, global warming is predicted to increase the frequency and severity of extremely HT in tropical and subtropical regions, and HT during grain filling has become one of the most constraint factors for the improvement of rice grain quality (Chaturvedi et al., 2017).

Nitrogen (N) fertilizer application is an important agronomic measure that affects rice growth, yield, and grain quality. N-deficiency reduced chlorophyll content and net photosynthetic rate in plant leaves and accelerated leaf senescence (Gu et al., 2015). On the other hand, heavy N supply extended the longevity of functional leaves and delayed leaf senescence (Singh et al., 2011; Wei H. H. et al., 2018). During the past few decades, large quantities of N fertilizer have been applied to maximize rice yields (Zhou et al., 2020). However, excessive use of N fertilizer resulted in a reduction of nitrogen utilization efficiency and environmental problems (Zhou et al., 2020). The previous studies have revealed that N fertilizer has a marked influence on protein content in rice grains, with the topdressing of N fertilizer at the panicle initiation stage (panicle N fertilizer) showing larger effect (Ning et al., 2020). The effects of N fertilizer on the appearance quality of rice grains were greatly variable, depending on the amount and timing of N fertilizer application (Wei H. Y. et al., 2018). For instance, an appropriate amount of fertilizer N application at the panicle initiation stage decreased the rate of chalky kernels, but the heavy application of N fertilizer, such as higher than 300 kg N ha^–1^, evidently increased the occurrence rate of chalky grains and thus undesirable grain appearance (Zhou et al., 2015). Nevertheless, the conflicting results are obtained for the effect of N fertilizer on grain starch component and relevant physicochemical parameters, e.g., the particle distribution of starch granule, thermal, and pasting properties (Singh et al., 2011; Gu et al., 2015; Zhu et al., 2016). Furthermore, several recent studies investigated the effect of two factors (elevated CO_2_ concentration and atmosphere temperature) on rice yield and grain quality under warming climate conditions (Madan et al., 2012; Jing et al., 2021). These works illustrated that the elevated CO_2_ concentration could deteriorate the negative impact of higher temperature on rice grain quality (Madan et al., 2012; Jing et al., 2021). Recently, Tang et al. (2019) revealed that an appropriate increase in N fertilizer application at panicle initiation effectively alleviated the negative effect of elevated temperature (ET) on grain quality by affecting grain starch physicochemical properties (Dou et al., 2017; Tang et al., 2019). The moderate application of N fertilizer was favorable for the improvements of rice yield and grain quality under the shading growth because N fertilizer significantly enhanced the activities of anti-oxidative enzymes both in leaves and in developing grains under shading growth (Wei H. Y. et al., 2018). However, less is known about the combined effect of N fertilizer application and HT on grain quality and its relation to the starch physicochemical properties and storage protein composition in rice grains.

In this article, two *japonica* rice cultivars being extensively planted in rice production were utilized to compare the variation of grain quality traits among different nitrogen-temperature treatments, with three N levels and two temperature regimes being designed. The objective of our work was to investigate the combined effects of N fertilizer and HT during grain filling on chalky occurrence, milling process, cooking and eating quality, as well as its relation to the particle distribution of starch granule and other relevant physicochemical parameters. Such results shall provide helpful knowledge for improving grain quality under the encounter of HT during grain filling by proper application of N fertilizer.

## Materials and Methods

### Plant Materials and Experimental Design

The experiment was conducted at the experimental station of Zijingang campus at the experimental station of Zijingang campus (30°18′N, 120°04′E) Zhejiang University in Hangzhou, China. A number of two lowland *japonica* rice cultivars (Xiushui 134 and Xiushui 09) with a large area of cultivation in the lower reaches of the Yangtze River, China were used in this study. Rice seeds were sown on seedling beds after indoor pre-germination on 20 May. The 25-day-old seedlings were subsequently transplanted into the plastic pots of quadrate shape (26 cm × 26 cm in area and 30 cm in height) filled with 15 kg of dry soil for each pot. A number of two plants were placed in each pot. The soil used in the plastic pots was air-dried, fully mixed, and then soaked before rice transplantation. The soil type in pots was yellow loam with a pH of 6.78, soil organic matter is 20.47 g kg^–1^, with 0.12% total nitrogen, 86.40 mg kg^–1^alkali-hydrolyzable N, 35.12 mg kg^–1^ available P, and 96.75 mg kg^–1^ exchangeable K. The amount of basal fertilizer was applied before rice transplanting, with 2.0 g urea and 1.0 g KH_2_PO_4_ for each pot. At 10th day after rice transplanting, 0.5 g urea was subsequently added to each pot as the tiller N topdressing. The same amount of basal and tiller fertilizers was considered for all the experimental pots until these pots were divided into three groups thereafter. Then, three N treatments were conducted as follows: (1) low nitrogen (LN): no panicle nitrogen fertilizer was added to the pot (zero panicle nitrogen fertilizer) after the applications of basal and tiller fertilizer; (2) moderate panicle nitrogen (MN): 1.0 g urea was added to each pot (approximately 40 kg ha^–1^) as panicle nitrogen fertilizer; (3) heavy panicle nitrogen (HN): 2.0 g urea was added to each pot (approximately 80 kg ha^–1^) as panicle nitrogen fertilizer. The panicle nitrogen fertilizer was applied at the panicle initiation stage (1.2th–1.5th leaf age in reverse order). All the pots were placed in a greenhouse under the natural light condition and moderate growth temperatures (28°C daytime/22°C nighttime) until rice plants were further imposed to different temperature treatments. Rice plants grown in different pots inside the greenhouse were usually irrigated every 2–3 days, depending on the requirements. Pest and disease were intensively controlled according to the locally recommended agronomic practices.

To evaluate the individual and interactive effects of air temperature and nitrogen application, the pots for the same N level of each rice cultivar were randomly classified into two groups. Then, the rice plants were moved into two phytotrons to impose different temperature treatments at the full heading stage (fourth day after anthesis). Different temperature regimes were performed using two phytotrons (Model PGV-36; Conviron, Winnipeg, MB, Canada). One phytotron was designed for the HT regime and the other for normal temperature (NT) control. The daily mean temperatures were 32°C for HT and 23°C for NT. The diurnal temperature change was designed by a simulation of daily temperature fluctuation based on the natural climate. The daily maximum and minimum temperatures were set up at 2:00 p.m. and 5:00 a.m., with 36 and 28°C for HT, 26 and 20°C for NT, respectively. For each genotype, four pots (two plants per pot) were imposed to HT, and another four pots were imposed to NT. Then, two phytotrons are kept without distinction in other climate conditions except temperature treatment. The photoperiod was from 5:30 a.m. to 7:00 p.m. with 160–190 J m^–2^ s^–1^ of light intensity, and the relative humidity was maintained around 75–80% with a wind speed of 0.5 m. s^–1^. A number of six treatments were as follows: (i) LN application under normal temperature (LN-NT); (ii) LN application under high temperature (LN-HT); (iii) MN application under normal temperature (MN-NT); (iv) MN application under high temperature (MN-HT); (v) HN application under normal temperature (HN-NT); and (vi) HN application under high temperature (HN-HT). Rice grain samples were harvested at maturity and then were air-dried at room temperature for 2 months before shelling and milling. The traits of milled quality and appearance quality were determined following the method described by Wei H. Y. et al. (2018). Afterward, rice grains were milled using a Satake rice machine (Satake Corp., Japan) and dried over a 200-mesh sieve. Rice flour was stored at −4°C for the further analysis of physical and chemical properties.

### Starch Isolation and Granule Size Distribution

Starch granule size distribution was determined using a laser particle diameter analyzer (Model LS 13320, Beckman Coulter, United States) according to the procedures of Liu et al. (2017). Before analysis, the starch samples were dispersed in 5 ml of 0.1 mol/L NaCl solution overnight. The solution was ground in a mortar with a pestle and filtered through a 74-mm sieve. The starch pellet was successively washed with 2 ml of 2 mol/L NaCl solution, 1 ml of 2% SDS, and 1 ml distilled water and centrifuged at 10,000 *g* for 10 min three times. Granule size expressed as average diameter was automatically calculated from instrument software.

### Observation of Starch Granule Morphology

The morphology of the starch granules was observed using scanning electron microscopy (SEM) with a secondary electron mode at 15 kV (JEOL-5600, Tokyo, Japan). Dried rice seeds were cut across the short axis with a razor blade to observe cross-sections of endosperm. The surface was sputter-coated with gold for SEM examination as described previously by Zhu et al. (2016).

### Starch Isolation and Granule Size Distribution

The swelling power (SP) of starches in urea solution was measured following the method described by Liu et al. (2017). About 20 mg of flour sample was dissolved at a series of concentrations of urea solution (0–9 M).

Starch thermal properties were determined using differential scanning calorimetry (DSC, Model DSC-7, Perkin-Elmer, Norwalk, CT, United States) equipped with an intra-cooling II system. Rice flour (2.5 mg) was equilibrated for 1 h before analysis. The sample was weighed in hermetic aluminum pans, and distilled water was added to obtain a flour-to-water ratio of 1:2 (w/w). The measurements were heated from 40 to 110°C at a heating rate of 10°C/min. The onset temperature of gelatinization (T_o_), peak temperature of gelatinization (T_p_), and conclusion temperature of gelatinization (T_c_) were determined from DSC thermograms as described by Zhong et al. (2005). Enthalpy (ΔH) was estimated by integrating the area between the thermogram and a baseline under the peak and was expressed as Joules per unit weight of dry starch (J/g).

### Determinations of Starch Pasting Properties

Pasting properties of rice starch were measured by a rapid viscosity analyzer (Model 3D, Newport Scientific, Australia) with Thermocline for Windows software (version 2.0). Rice flour (3 g each sample) was mixed with 25 ml of distilled water in an aluminum RVA canister. A programmed heating and cooling cycle were set as described by Zhong et al. (2005). The starch sample was held at 50°C for 1 min, heated from 50 to 95°C at 12°C/min, held at 95°C for 2.5 min, cooled to 50°C at 12°C/min, and held at 50°C for 2.5 min. All measurements were two times replicated. The peak viscosity (PKV), hot paste viscosity (HPV), cold paste viscosity (CPV), and their derivative parameters, including breakdown (BD = PV-HPV) and setback (SB = CPV-PV), were recorded. Triplicate measurements were taken for each sample.

### Chemical Analysis of Starch Components and Protein Content

Amylose content (AC) was measured by iodine colorimetry method based on the procedure of Zhong et al. (2005); blue value (BV) and maximum absorption (λ_max_) of amylose and amylopectin were determined according to the method of Liu et al. (2017), and a modified alkaline steeping method was used to fractionate and isolate starch before measurement. The isolated amylose and amylopectin powers were separately diluted with distilled water and performed the iodine reaction. The absorbance spectrum of the starch-iodine complex was recorded from 500 to 700 nm, and the wavelength of maximum absorption was determined. BV was the absorbance at 600 nm for amylose and at 680 nm for amylopectin, respectively.

Total protein content in milled rice flour was determined according to Martin and Fitzgerald (2002) using the Kjeldahl method. A number of four protein fractions were sequentially extracted in the order below by stirring the flour (0.5 g flour/25 ml solvent) for 2 h at room temperature in the following solvents. For albumin: 10 mM Tris–HCl, pH 7.5; for globulin: 1 M NaCl, 10 mM Tris–HCl, pH 7.5; for prolamin: 55% (v/v) *n*-propanol, 10 mM Tris–HCl, pH 7.5; and for glutelin: 0.24% CuSO_4_, 1.68% KOH, 0.5% potassium sodium tartrate, and 50% (v/v) *iso*-propanol. The extraction for glutelin is identical to the biuret reagent. After centrifugation at 4,000 *g* for 10 min at room temperature, the contents of the four protein fractions were determined as previously reported by Liu et al. (2005).

### Statistical Analysis

Data analysis was performed using a statistic software version SPSS 16.0 (SPSS, Inc., Chicago, IL, United States). The data were submitted to variance analysis, and the means were tested by least significant difference (LSD) at 5% probability. At least three biological replicate samples were used for three technical determinations to characterize the samples.

## Results

### Effect of Nitrogen-Temperature Treatments on Milling Quality and Appearance Quality

As shown in Table 1, the application of panicle N fertilizer (both MN and HN) tended to enhance the values of brown grain rate (BR), milled grain rate (MR), and head milled grain rate (HMR), while HT exposure evidently deteriorated the milling quality of rice grains, with the significantly lower BR, MR, and HMR for HT relative to NT under the same N level. In contrast, HT exposure appeared to have a more considerable impact on milling quality than N fertilizer. However, the dropping extent of BR, MR, and HMR under HT exposure was partially attenuated by panicle N fertilizer application, with the significantly higher values of MR and HMR for MN-HT and HN-HT relative to LN-HT. This result indicated clearly that the increasing application of N fertilizer (MN and HN) had a positive impact on milling quality for rice plants being imposed to HT regime.

**TABLE 1:** Difference in milling property and appearance quality among different treatments.

Cultivars	Treatments		Brown grain rate (%)	Milled grain rate (%)	Head milled grain rate (%)	Grain chalky rate (%)	Grain chalky degree (%)	L/B ratio
	Nitrogen levels	Temperature regimes						
Xiushui134	LN	NT	80.20b	73.26b	62.85b	22.30c	7.95c	1.47a
		HT	75.67d	61.13e	45.72d	79.83a	57.84a	1.49a
	MN	NT	81.52a	74.38a	65.27a	9.75d	5.94c	1.51a
		HT	78.71c	67.47c	51.32c	59.50b	46.53b	1.48a
	HN	NT	81.34a	73.86ab	62.74b	25.92c	8.38c	1.52a
		HT	76.23cd	64.36d	51.18c	85.71a	60.67a	1.49a
Xiushui09	LN	NT	83.11b	67.46b	61.33b	17.91d	8.30d	1.56a
		HT	68.17d	54.50d	44.71d	56.74b	47.43b	1.53a
	MN	NT	86.69a	70.63a	66.87a	10.69e	4.56e	1.60a
		HT	78.66c	59.65c	50.56c	42.33c	35.87c	1.58a
	HN	NT	82.34b	67.87b	63.92ab	16.62d	7.58d	1.62a
		HT	77.62c	60.18c	48.33c	90.92a	58.55a	1.59a
Nitrogen			53.56**	22.30**	33.50**	258.47**	75.74**	2.90ns
Temperature			336.82**	422.92**	973.23**	3,550.54**	3,784.68**	3.83*
Nitrogen × temperature			17.61**	4.13*	6.70**	80.77**	40.11**	0.23ns

*LN, MN, and HN indicate low nitrogen, moderate nitrogen, heavy nitrogen, respectively; NT and HT indicate normal temperature and high temperature, respectively. L/B ratio, ratio of grain length to width.*

Data followed by the same small letter in the same column indicate insignificant difference (p < 0.05) between different treatments for the same rice cultivar. *, ** indicates the significance at 0.05 and 0.01 probability level, respectively.

For appearance quality, no significant difference in length/width ratio was found among six treatments, while the effects of N levels, temperature, and their interaction on chalky grain rate (CGR, %) and chalky grain degree (CGD, %) were statistically significant (Table 1). Comparatively, the CGR and CGD of three HT regimes (LN-HT, MN-HT, and HN-HT) were 3.3–7.8-fold higher than those of three NT counterparts (LN-NT, MN-NT, and HN-NT), indicating that the CGR and CGD of milled grains were significantly enhanced by HT exposure, irrespective of N levels. For the two rice cultivars, the orders of CGR and CGD for six treatments were as follows: MN-NT < LN-NT and HN-NT < MN-HT < HN-HT and LN-HT (Table 1). The lower CGD under MN-HT relative to LN-HT implied that moderate amount of N fertilizer application (MN) could be partially inhibited or mitigated the HT-induced increase in chalky occurrence. Different from the variation in grain milling quality (BR, MR, and HMR) among six treatments, the CGR and CGD under HN-HT appeared to be higher than those under LN-HT (Table 1). This phenomenon implied that excessive N fertilizer application possibly exacerbated HT-induced enhancement in CGR and CGD, rather than an inhibitory effect on HT-induced chalky occurrence.

### Effect of Nitrogen-Temperature Treatments on Starch Granule Size Distribution

**FIGURE 1:**
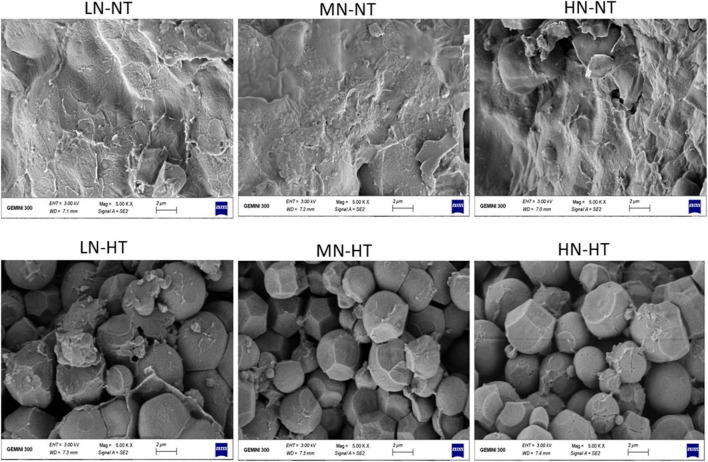
Comparison of starch granule morphology for six treatments by using SEM. LN-NT, MN-NT, HN-NT, LN-HT, MN-NT, and HN-HT indicate low nitrogen-normal temperature, moderate nitrogen-normal temperature, high nitrogen-normal temperature, low nitrogen-high temperature, moderate nitrogen-high temperature, and high nitrogen-high temperature, respectively.

Scanning electron microscopy imaging indicated that HT exposure largely affected the size and shape of starch granules as well as their space-packed structure, with the spherical shape starch granule and loosely packed granular structure with large air space being observed for HT-ripening grains. In contrast, the polyhedral shape and densely packed starch granular structure were visually observed for NT ripening grains, irrespective of N levels. On the other hand, only a slight difference in starch granule morphology and their space-packed structure was found among different N levels using SEM observation (Figure 1).

**FIGURE 2:**
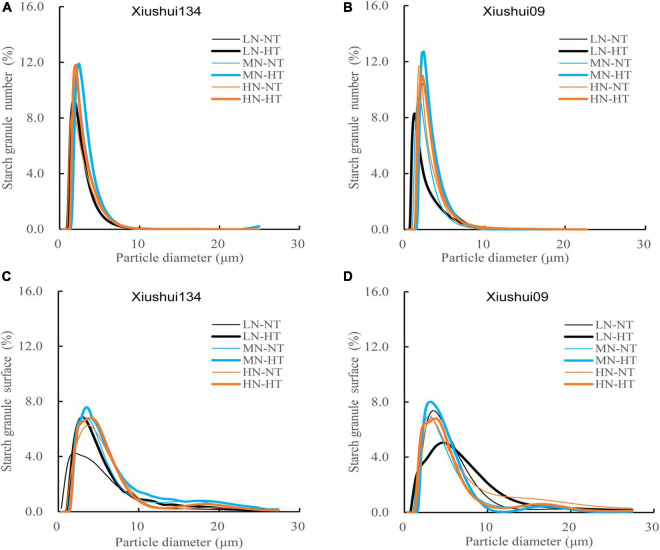
Comparison of starch particle distribution (number- and surface area based proportion) for different nitrogen-temperature treatments. LN-NT, MN-NT, HN-NT, LN-HT, MN-NT, and HN-HT indicate low nitrogen-normal temperature, moderate nitrogen-normal temperature, high nitrogen-normal temperature, low nitrogen-high temperature, moderate nitrogen-high temperature, and high nitrogen-high temperature, respectively. **(A)** Starch particle distribution of percentage number in Xiushui134, **(B)** Starch particle distribution of percentage number in Xiushui09, **(C)** Starch particle distribution of percentage surface in Xiushui134, **(D)** Starch particle distribution of percentage surface in Xiushui09.

We further investigated the difference in the particle distribution of starch granules among six treatments (Figure 2). Particle size distributions of rice flours exhibited a unimodal profile, with the shifted peak positions and its height of particle distribution profile for six treatments. In contrast, HT exposure evidently enhanced the average diameter of starch granules and decreased the number-based percentage of smaller size starch granules (Figures 2A,B). For the surface area-based percentage of starch granules, the effects of HT exposure on the surface area-based percentage were somewhat variable, depending on rice cultivars and N levels (Figures 2C,D). For the three N levels, HT-induced alteration in the surface area-based distribution was much larger for LN relative to MN and HN levels, with a more remarkable change in the profile of particle size distribution between LN-HT and LN-NT than those between MN-HT and MN-NT, and between HN-HT and HN-NT (Figures 2C,D). These results indicated that the application of N fertilizer disturbed the effect of HT exposure on the particle size distributions of starch granules of the number- and surface area-based proportions.

Table 2 presented the variation in the number, volume, and surface area, percentage of starch granule distribution among six treatments, with rice starch granules being further divided into three size classes: small starch granule (D ≤ 2.0 μm), medium starch granule (2.0 μm < D ≤ 8.0 μm), and large starch granule (D > 8.0 μm) as described previously by Zhu et al. (2017). The result showed that the number-based percentages of medium starch granules (2.0 μm < D ≤ 8.0 μm) under three HT regimes (LN-HT, MN-HT, and HN-HT) were significantly higher than those of their NT counterparts (LN-NT, MN-NT, and HN-NT), with the opposite change for the number-based percentages of smaller starch granules (D ≤ 2.0 μm) (Table 2). These results implied that HT exposure might promote the smaller starch granule to develop or extend into the medium-sized starch granules (2.0 μm < D ≤ 8.0 μm), due to the simultaneous increments in the number and surface area proportions of medium starch granules (2.0 μm < D ≤ 8.0 μm) for MN-HT and HN-HT than LN-HT. For three N levels, MN had a relatively narrower change in the number- and surface area-based percentages of medium size starch granules (2.0 μm < D ≤ 8.0 μm) between the two temperature regimes (NT and HT) than LN and HN. Variation analysis indicated that the interactive effect between N level and ambient temperature on the number, volume, and surface area percentages of starch granule distribution was statistically significant for all the three types of starch granules (Table 2).

**TABLE 2:** Difference in starch granule distribution (by number-, volume-, and surface-based percentages) among different treatments.

Cultivars	Treatments		D ≤ 2.0 μm			2.0 μm < D ≤ 8.0 μm			D > 8.0 μm		
	Nitrogen levels	Temperature regimes	Number (%)	Volume (%)	Surface (%)	Number (%)	Volume (%)	Surface (%)	Number (%)	Volume (%)	Surface (%)
Xiushui134	LN	NT	44.89a	4.62b	15.48b	54.68d	45.39e	77.18c	0.43c	50.00a	7.34c
		HT	25.66c	2.63c	41.70a	73.55bc	62.79d	52.23d	0.79a	34.58b	6.07d
	MN	NT	28.86b	4.06b	5.19d	70.66c	79.22a	84.60ab	0.48c	16.72d	10.21a
		HT	25.12c	3.07c	7.60c	74.15b	69.71c	80.90b	0.73a	27.22bc	11.50a
	HN	NT	47.09a	5.24a	14.33b	52.31d	74.19b	77.12c	0.60b	20.57c	8.54b
		HT	11.04d	1.15d	5.80cd	88.63a	66.72cd	86.78a	0.33d	32.13b	7.42c
Xiushui09	LN	NT	64.95a	4.30b	18.27a	34.13e	45.53d	64.65d	0.92b	50.18b	17.08a
		HT	16.96d	1.55c	4.72d	82.12b	64.90c	85.48ab	0.92b	33.55c	9.80b
	MN	NT	43.68b	6.24a	15.25b	55.91d	74.20a	77.93c	0.41c	19.56e	6.82d
		HT	19.08d	1.28c	5.23d	79.76b	42.85d	77.08c	1.16a	55.86a	17.69a
	HN	NT	32.00c	4.26b	10.97c	67.59c	70.61b	81.58b	0.41c	25.13d	7.45c
		HT	7.51e	0.74d	2.29e	92.07a	63.66c	90.69a	0.42c	35.60c	7.02cd
Nitrogen			229.10**	12.18**	398.05**	200.90**	203.51**	138.79**	214.64**	154.46**	330.75**
Temperature			2,402.79**	511.30**	26.80**	1,990.43**	10.52**	25.25**	30.38**	76.76**	10.31**
Nitrogen × temperature			127.83**	9.52**	126.71**	112.57**	344.09**	28.87**	184.43**	276.85**	577.46**

*LN, MN, and HN indicate low nitrogen, moderate nitrogen, heavy nitrogen, respectively; NT and HT indicate normal temperature and high temperature, respectively.*

Data followed by the same small letter in the same column indicate insignificant difference (p < 0.05) between different treatments for the same rice cultivar; *, ** indicates the significance at 0.05 and 0.01 probability level, respectively.

### Effect of Nitrogen-Temperature Treatments on Amylose Content and Starch Components

From Table 3, the AC of milled rice grains was largely affected by both ambient temperature and N levels. In generally, HT exposure resulted in much more enlarged alteration in AC than N levels. Interestingly, HT exposure reduced AC (%) for all the three N level, while the effect of N fertilizer on AC (%) was greatly variable, depending on environmental temperature. For instance, HN had relatively lower AC than LN for HT-ripening grains, while HN had relatively higher AC than LN under NT growth. Thus, AC was highest for HN-NT and lowest AC for HN-HT among six treatments (Table 3), implying that heavy application of N fertilizer (HN) might widen or enlarge the variability of AC between HT and NT.

**TABLE 3:** Difference in chemical component related to cooking and eating quality among different treatments.

Cultivars	Treatments		Starch (mg/g)	AC (%)	GC (mm)	Am		Ap	
	Nitrogen levels	Temperature regimes				BV (Am)	λ (Am) (nm)	BV (Ap)	λ (Ap) (nm)
Xiushui134	LN	NT	830.70a	18.66ab	72.34b	0.384a	610.0a	0.135a	520.0b
		HT	816.44b	15.69c	67.75c	0.225e	590.2c	0.116d	516.7bc
	MN	NT	820.54bc	18.38b	74.66a	0.356b	600.0b	0.131ab	505.6c
		HT	799.65c	14.40d	67.38cd	0.281cd	588.9cd	0.120cd	530.0a
	HN	NT	810.48d	19.03a	74.28a	0.296c	600.0b	0.127b	501.3d
		HT	769.74e	12.82e	66.81d	0.279d	582.7d	0.124c	526.7ab
Xiushui09	LN	NT	803.26a	16.85b	80.52b	0.287a	600.0a	0.109bc	524.5a
		HT	774.73b	15.23c	73.44c	0.210c	573.3c	0.095d	510.3c
	MN	NT	795.10b	16.64b	81.28a	0.246b	590.0b	0.111b	520.0ab
		HT	779.65c	14.18d	73.61c	0.168d	560.0d	0.106c	516.8b
	HN	NT	793.15d	17.62a	80.36b	0.203c	576.7c	0.120a	526.7a
		HT	767.69e	13.93d	70.32d	0.167d	560.0d	0.113b	517.8b
Nitrogen			89.06**	9.12**	4.47*	62.26**	5.61*	11.43**	9.12**
Temperature			345.74**	464.53**	433.47**	616.76**	7,092.91**	70.57**	19.05**
Nitrogen × temperature			12.13**	23.05**	5.71*	79.23**	56.07**	7.71*	12.43**

*LN, MN, and HN indicate low nitrogen, moderate nitrogen, heavy nitrogen, respectively; NT and HT indicate normal temperature and high temperature, respectively.*

*AC, amylose content; BV_Am_ and λmax, blue value and maximum absorbance of amylose; BV_Ap_ and λmax, blue value and maximum absorbance of amylopectin; GC, Gel consistency.*

Data followed by the same small letter in the same column indicate insignificant difference (p < 0.05) between different treatments for the same rice cultivar; *,** indicates the significance at 0.05 and 0.01 probability level, respectively.

**FIGURE 3:**
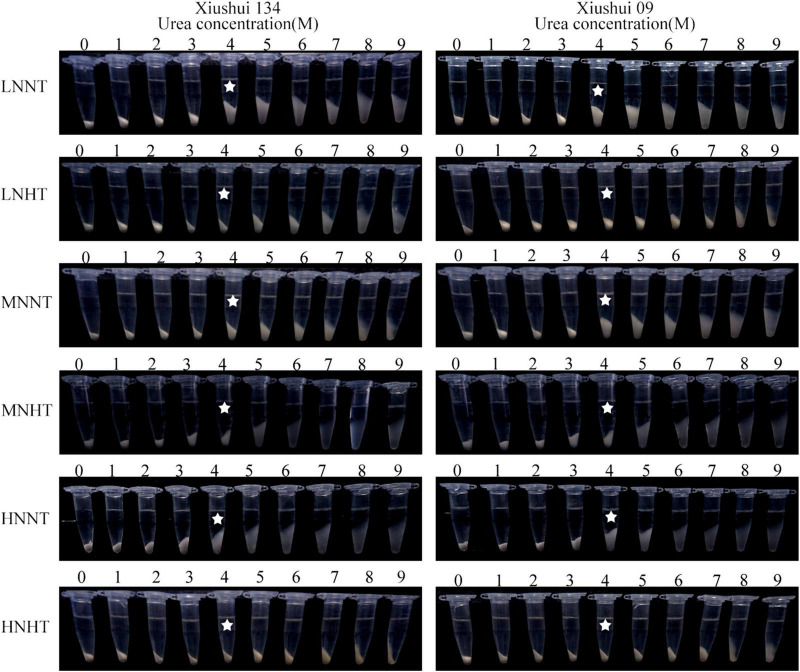
Effects of the urea concentrations on SP of starch granules for different nitrogen-temperature treatments. LN-NT, MN-NT, HN-NT, LN-HT, MN-NT, and HN-HT indicate low nitrogen-normal temperature, moderate nitrogen-normal temperature, high nitrogen-normal temperature, low nitrogen-high temperature, moderate nitrogen-high temperature, and high nitrogen-high temperature, respectively.

Just like AC variation among six treatments, HT exposure had a more considerable impact on GC, and BV, and maximum absorbance (λmax) of amylose and amylopectin components than N fertilizer (Table 3). Comparatively, the GC and BV (Am) of HT-ripening grains were significantly lower than those of their NT counterparts for all the three N levels, while the λmax (Ap) of amylopectin was, to some extent, enhanced by HT exposure (Table 3). Interestingly, MN-HT tended to have higher GC values than HN-HT, but the GC values under MN-HT still were much lower than those of all the NT-ripening grains (Table 3). Hence, the positive effect of moderate N level (MN) on GC under HT exposure was insufficient to compensate for the markedly lowered extent of GC value in HT-ripening grains. The SP experiment indicated that HT-ripening grains tended to be more resistant to 4 M concentration urea than their NT-ripening counterparts, while only slight alteration was observed in SP among the three N levels under the same temperature regime (Figure 3). This result suggested that HT-ripening grains had lower starch digestibility values and higher GT than NT-ripening grains, irrespective of different N levels.

### Effect of Nitrogen-Temperature Treatments on Starch Thermal and Pasting Properties

**FIGURE 4:**
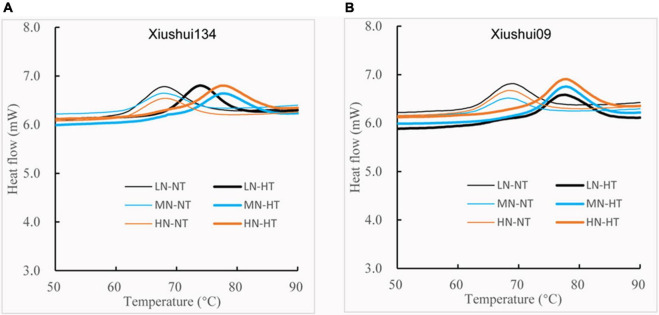
Comparison of DSC profile for different nitrogen-temperature treatments. LN-NT, MN-NT, HN-NT, LN-HT, MN-NT, and HN-HT indicate low nitrogen-normal temperature, moderate nitrogen-normal temperature, high nitrogen-normal temperature, low nitrogen-high temperature, moderate nitrogen-high temperature, and high nitrogen-high temperature, respectively. **(A)** DSC profile of Xiushui134; **(B)** DSC profile of Xiushui09.

The thermal property of rice starch varied strikingly between NT and HT, with the relatively small change in DSC curves for three N levels at the same temperature regime (Figure 4). This result indicated clearly that HT exposure had a more profound impact on the starch thermal property than N level. For a comparison of DSC parameters among three N levels under NT regime, LN-NT tended to have relatively higher Tp and gelatinization enthalpy (ΔH) than MN-NT and HN-NT, but it was this case for HT regime, the order of ΔH was: HN-HT > MN-HT > LN-HT among three HT regimes, indicating that both HN-HT and MN-HT had significantly higher ΔH values than LN-HT (Table 4). In this regard, the influence of N fertilizer on To, Tp, Tc, and ΔH showed the reverse tendencies under HT and NT. Variation analysis showed that the individual and interactive effects of the two factors (N fertilizer and temperature) on starch gelatinization transition temperature (To, Tp, and Tc) and ΔH were statistically significant (Table 4). We suggested that N fertilizer application exacerbated the deteriorative impact of HT exposure on cooking and eating quality by additively enhancing starch gelatinization enthalpy, because the higher GT and ΔH were the indicators for the increased energy requirement and longer cooking time in the starch gelatinization process.

**TABLE 4:** Difference in DSC parameters among different treatments.

Cultivars	Treatments		T_o_ (°C)	T_p_ (°C)	T_c_ (°C)	ΔH (J/g)
	Nitrogen levels	Temperature regimes				
Xiushui134	LN	NT	61.16d	67.37c	74.51d	11.62d
		HT	67.51b	73.27b	79.01c	13.37c
	MN	NT	61.26d	67.50c	74.46d	11.35d
		HT	70.14a	77.01a	84.49b	13.92b
	HN	NT	62.17c	67.56c	73.87e	10.78e
		HT	69.72a	77.31a	85.18a	14.27a
Xiushui09	LN	NT	62.23b	68.22b	74.38c	10.34d
		HT	71.80a	77.11a	83.67b	11.83c
	MN	NT	61.32c	67.14b	74.05d	10.02e
		HT	71.72a	77.26a	83.98ab	12.75b
	HN	NT	61.96bc	67.93b	74.16cd	10.23de
		HT	71.58a	77.34a	84.26a	13.37a
Nitrogen			14.23**	27.81**	208.19**	14.89**
Temperature			6809.44**	5808.74**	19728.34**	2035.34**
Nitrogen × temperature			21.48**	43.16**	318.66**	77.41**

*LN, MN, and HN indicate low nitrogen, moderate nitrogen, heavy nitrogen, respectively; NT and HT indicate normal temperature and high temperature, respectively.*

*T_o_, onset temperature of gelatinization; T_p_, peak temperature of gelatinization; T_c_, conclusion temperature of gelatinization; ΔH, enthalpy.*

Data followed by the same small letter in the same column indicate insignificant difference (p < 0.05) between different treatments for the same rice cultivar; *, ** indicates the significance at 0.05 and 0.01 probability level, respectively.

**FIGURE 5:**
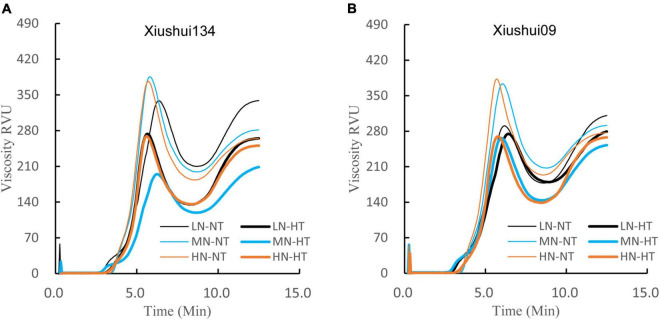
Comparison of RVA profile for different nitrogen-temperature treatments. LN-NT, MN-NT, HN-NT, LN-HT, MN-NT, and HN-HT indicate low nitrogen-normal temperature, moderate nitrogen-normal temperature, high nitrogen-normal temperature, low nitrogen-high temperature, moderate nitrogen-high temperature, and high nitrogen-high temperature, respectively. **(A)** RVA profile of Xiushui134; **(B)** RVA profile of Xiushui09.

Figure 5 presented the difference in starch RVA profile among six treatments. The result showed that the RVA profile of rice starch was evidently affected by both N fertilizer and ambient temperature. Under NT condition, MN and HN had the relatively higher PKV and lower CPV than LN (Figure 5). For the same N level, HT exposure decreased PKV, HPV, and CPV (Table 5). However, the effect of HT exposure on BD and setback was somewhat variable among different N levels, with the significantly lower BD and a higher setback for MN-HT and HN-HT compared to their NT counterparts under the same N level (MN-NT and HN-NT) (Table 5). Considering the fact that the rice varieties with superior palatability generally had a higher BD and lower setback than those with inferior quality, we proposed that N fertilizer application exacerbated the HT-induced deterioration in cooking and eating quality, in terms of the relatively lower BD and higher setback value for MN-HT and HN-HT compared with LN-HT (Table 5).

**TABLE 5:** Difference in RVA parameters among different treatments (RVU).

Cultivars	Treatments		PKV	HPV	Breakdown	CPV	Setback
	Nitrogen levels	Temperature regimes					
Xiushui134	LN	NT	338.67b	132.83c	205.83a	338.87a	0.25b
		HT	273.83c	135.42c	138.42c	264.92c	−8.92b
	MN	NT	385.67a	199.33a	186.33b	281.33b	−104.33d
		HT	194.75d	77.67d	117.08d	208.58e	13.83a
	HN	NT	376.58a	183.33b	193.25ab	264.42c	−112.17d
		HT	269.42c	135.83c	133.58c	250.58d	−18.83c
Xiushui09	LN	NT	290.67b	177.75c	112.92d	310.92a	20.25a
		HT	274.92c	179.75c	95.17e	279.25c	4.33b
	MN	NT	373.58a	207.50a	166.08b	291.33b	−82.25d
		HT	266.42d	143.75d	122.67cd	252.50e	−13.92c
	HN	NT	382.75a	193.67b	189.08a	276.67c	−106.08e
		HT	269.83cd	139.17d	130.67c	267.83d	−2.00b
Nitrogen			116.41**	22.22**	64.76**	215.20**	1,344.85**
Temperature			3,722.21**	2,821.56**	962.68**	557.61**	3,182.73**
Nitrogen × temperature			378.15**	959.94**	9.02**	71.80**	1,166.78**

*LN, MN, and HN indicate low nitrogen, moderate nitrogen, heavy nitrogen, respectively; NT and HT indicate normal temperature and high temperature, respectively.*

*PKV, peak viscosity; HPV, hot paste viscosity; CPV, cool paste viscosity; BD, breakdown; SB, setback.*

Data followed by the same small letter in the same column indicate insignificant difference (p < 0.05) between different treatments for the same rice cultivar; *, ** indicates the significance at 0.05 and 0.01 probability level, respectively.

### Effect of Nitrogen-Temperature Treatments on Protein Content and Its Compositions

The total protein content in milled grains was largely affected by N fertilizer and HT as well as their interaction (*p* < 0.05) (Table 6). In general, MN and HN had significantly higher grain protein content than LN. Under the same N level, the total protein content of HT-ripening grains was significantly higher than that of their NT counterparts. For all the six treatments, the total protein content in milled rice grains is highest for HN-HT and lowest for LT-NT, respectively (Table 6). Hence, the effects of higher N fertilizer and HT exposure on grain total protein content appeared to be additively enhanced. Considering the inferior palatability performance being often caused by the excessively higher protein content in rice grains (Fitzgerald et al., 2009), we proposed that the increased N fertilizer application should exacerbate the negative influence of HT exposure on the palatability of cooking rice, rather than an alleviation for the HT-induced deterioration in cooking and eating quality.

**TABLE 6:** Difference in total protein content and its four compositions among different treatments.

Cultivars	Treatments		TPC (%)	Albumin (%)	Globulin (%)	Prolamin (%)	Glutelin (%)	Glutelin/prolamin
	Nitrogen levels	Temperature regimes						
Xiushui134	LN	NT	8.67d	0.47c	0.45d	0.65c	7.48d	11.51b
		HT	10.03b	0.49c	0.46d	0.68bc	8.31b	12.22a
	MN	NT	9.75bc	0.53b	0.51c	0.72b	8.12bc	11.28c
		HT	10.52a	0.61a	0.63a	0.74b	8.65a	11.69b
	HN	NT	9.64c	0.52bc	0.52c	0.81a	8.04c	9.92d
		HT	10.48a	0.55b	0.56b	0.79a	8.76a	11.09c
Xiushui09	LN	NT	8.16d	0.35d	0.31c	0.65c	6.93d	10.66b
		HT	10.11b	0.44c	0.38c	0.69c	8.68a	12.58a
	MN	NT	8.87c	0.43c	0.39c	0.78b	7.37c	9.44c
		HT	10.68a	0.62a	0.58ab	0.81b	8.58a	10.59b
	HN	NT	9.95b	0.54b	0.56b	0.93a	7.91b	8.51d
		HT	10.91a	0.59a	0.61a	0.95a	8.71a	9.17c
Nitrogen			33.62**	122.50**	56.06**	13.98**	117.72**	89.77**
Temperature			155.62**	80.00**	13.92**	150.73**	83.17**	372.95**
Nitrogen × temperature			13.77**	17.97**	13.56**	14.62**	13.77**	15.62**

*LN, MN, and HN indicate low nitrogen, moderate nitrogen, heavy nitrogen, respectively; NT and HT indicate normal temperature and high temperature, respectively.*

*TPC, total protein content; the values of TPC were not estimated by the sum of four compositions.*

TPC, total protein content; data followed by the same small letter in the same column indicate insignificant difference (p < 0.05) between different treatments for the same rice cultivar; *, ** indicates the significance at 0.05 and 0.01 probability level, respectively.

Nitrogen fertilizer and HT exposure evidently increased the glutelin content in milled grains, with an additive increase in glutelin content for HN-HT than LN-NT. However, it appeared to be not this case for prolamin content (Table 6). Thus, HT exposure significantly enhanced the ratio of glutelin to prolamin (glutelin/prolamin) in milled rice grains, while the ratio of glutelin to prolamin in milled grains kept relatively stable among the three N levels under the same temperature. Comparatively, the contents of both prolamin and glutelin tended to increase concurrently with the increased level of N fertilizer (Table 6). Considering the increased glutelin or prolamin in HT-ripening grains, we inferred that HT-induced deterioration in cooking and eating quality possibly was not attributable to the adverse influence of prolamin on the palatability of cooking rice. Instead, the additive increases in grain total protein and glutelin contents might be, at least partly, responsible for the exacerbated deterioration in cooking and eating quality for rice plants being imposed to HT exposure under higher N level (HN-HT).

## Discussion

It had been well documented that the quality traits of rice grains depend on not only genetic background, but also on the climatic condition and agronomic management during growth and development (Fitzgerald et al., 2009). Among various environmental factors, the ambient temperature during grain-filling period and nitrogen fertilizer application was widely considered to be two of the most factors influencing rice yield and grain quality (Patindol et al., 2015; Cheng et al., 2019; Tang et al., 2019). HT exposure accelerated grain filling and shortened the duration of grain filling, thereby leading to insufficient starch accumulation and chalky occurrence for HT-ripening grains (Mitsui et al., 2016; Cheng et al., 2019), while the suitable application of panicle nitrogen fertilizer prolonged the lasting duration of grain-filling process and decreased the chalky occurrence of rice grains (Wei H. Y. et al., 2018), with the significant increase in chalky grain rate for both inadequate supply and overuse of nitrogen fertilizer (Yang et al., 2007; Wei H. Y. et al., 2018; Ning et al., 2020). In our present result, the orders of CGR and CGD for six treatments were: MN-NT < LN-NT and HN-NT < MN-HT < HN-HT and LN-HT, with the lowest CGR and CGD under MN-NT for the two rice cultivars (Table 1). This result confirmed that moderate amount of nitrogen fertilizer application (MN) effectively decreased the CGR and CGD values of rice grain and improved grain appearance quality under normal growth (HT), which was basically consistent with the reports of the previous studies (Yang et al., 2007; Wei H. Y. et al., 2018; Ning et al., 2020). For the combined effect of nitrogen fertilizer and HT on grain chalky occurrence, MN-HT was found to have the significant lower CGD than LN-HT (Table 1), implying that moderate amount of N fertilizer application (MN) could be partially inhibited the HT-induced increase in grain chalky occurrence. However, our present result revealed that heavy N fertilizer in combination with HT exposure (HN-HT) had significantly higher CGR and CGD than heavy N supply alone (HN-NT) (Table 1), and the tendency of difference between HN-HT and LN-HT in CGR and CGD was quite different from that in BR, MR, and HMR (Table 1). In this regard, excessive N fertilizer application exacerbated the HT-induced enhancement in CGR and CGD, rather than an inhibitory effect on HT-induced chalky occurrence. While for grain milling quality traits (BR, MR, and HMR), the increasing application of N fertilizer (MN and HN) had a positive impact on milling quality for rice plants being imposed to HT regime (Table 1). The reason could be partly explained by their difference in the sensitivity to environmental adversity between milling quality traits and chalky occurrence, because grain chalky occurrence is more easily influenced by HT and other environmental adversity than milling quality (BR, MR, and HMR) (Fitzgerald et al., 2009; Mitsui et al., 2016). In the previous studies, Tang et al. (2019) revealed that the increasing application of panicle N fertilizer effectively compensated for the negative effect of ET on grain chalky occurrence. However, our present result indicated that HT exposure had more considerable impact on chalky occurrence than the varying N levels, and the effect of the varying N levels on chalky occurrence and starch granule morphology and their packing structure was largely covered up by the larger effect of HT exposure on them. The possible reason for such the discrepant results was mostly caused by the severity of HT, N fertilizer level, and soil backgrounding.

Starch is the most predominant component, accounting for about 90% of dry matter of rice flour. Starch composition and granule structure play a dominant role in determining the physicochemical properties of rice flour (Fitzgerald et al., 2009). The previous studies had demonstrated that HT exposure during filling period significantly affected the AC and amylopectin fine structure of rice starch (Liu et al., 2017), concurrently with the increased GT value for HT-ripening grains (Zhong et al., 2005; Liu et al., 2017). However, the previous studies showed the conflicting reports for the variation in starch composition and its physicochemical properties in response to nitrogen fertilizer. For instance, Singh et al. (2011) reported that the higher level of N fertilizer significantly decreased the AC and GC of rice flour, while the opposite result was obtained by Ning et al. (2020), who concluded that AC tended to increase progressively with the increasing nitrogen level. Zhu et al. (2017) revealed that heavy N fertilizer significantly enhanced the SP, gelatinization enthalpy, and solubility of rice starch. Conversely, several other studies argued that the increasing level of N fertilizer decreased the gelatinization enthalpy and/or only slight changes in AC level and amylopectin composition (Yang et al., 2007). Nevertheless, these discordant phenomena did not exclude the possibility that the effect of N fertilizer on the starch component could be largely disturbed by the impact of varying temperature during filling period, because of HT exposure had much more striking impact on starch component and its morphological structure than the varying N fertilizer under the same temperature regime (Table 3 and Figures 2, 3). Furthermore, the effect of N fertilizer on AC (%) was greatly variable, depending on the environmental temperature. In our present results, HN had relatively lower AC than LN under HT exposure, while HN had relatively higher AC than LN under NT growth (Table 3). Among the six treatments, AC showed the highest for HN-NT and the lowest for HN-HT, respectively (Table 3). The effect of panicle N fertilizer on Tp and ΔH was largely disturbed by environmental temperature during filling period (Table 4). Hence, HT exposure in combination with heavy N fertilizer (HN-HT) decreased the AC level of milled grains more strikingly than HT alone (Table 3). In light of the fact that rice grains with lower AC generally had the superior palatability of cooking rice than those with higher AC (Bao, 2012), we inferred that the deteriorated palatability of HT-ripening grains (Fitzgerald et al., 2009) and the negative impact of heavier N fertilizer application on the palatability of cooked rice and the negative impact of heavier N fertilizer application on the palatability of cooked rice (Gu et al., 2015) were not mainly ascribed to the significantly lowered level of AC under HT exposure alone or/and its combination with heavy N application (HN-HT).

The palatability of cooked rice does not always depend on AC and GC, because the particle size distribution of starch granules contributed greatly to the cooking and eating quality by affecting starch GT and other physicochemical properties (Zhu et al., 2017). In our present result, the increasing level of N fertilizer application exacerbated the deteriorative impact of HT exposure on cooking and eating quality by additively enhancing starch gelatinization enthalpy (ΔH) (Table 4), which was coincident with the increase in the number- and surface area-based percentages of medium size starch granules (2.0 μm < D ≤ 8.0 μm) under HN-HT and MN-HT (Table 2). The previous studies had revealed that the higher temperature enhanced the average diameter of starch granules, but it lowered the total surface areas of starch granules, consequently leading to a marked enhancement in starch GT under HT exposure (Dou et al., 2017; Liu et al., 2017). However, our present result revealed that three HT regimes (LN-HT, MN-HT, and HN-HT) did not always show the dropping tendency in the surface area-based percentage of medium starch granules (2.0 μm < D ≤ 8.0 μm) in comparison with their NT counterparts (LN-NT, MN-NT, and HN-NT). For three N levels, MN had a relatively narrower change in the number- and surface area-based percentages of medium size starch granules (2.0 μm < D ≤ 8.0 μm) between the two temperature regimes than LN and HN (Table 2). The previous studies on wheat and other cereal crops had revealed that the large-size starch granules (LSGs) in wheat generally formed first at the early filling stage, whereas the small-size starch granules (SSGs) appeared late in kernel development (Bechtel et al., 1990; Lindeboom et al., 2004). The proliferation and development of SSGs might be more severely impaired by HT exposure relative to LSGs in amyloplasts, consequently the increased percentage of LSGs under HT exposure (Liu et al., 2011), because the limited substrate for starch accumulation was mainly partitioned for the formation of elongated starch granules and growing starch granules, not for the generation or proliferation of more newly starch granules and their number enlargement in amyloplasts (Geigenberger, 2011; Liu et al., 2011). Hence, application of N fertilizer under HT exposure showed the simultaneous increments in the number and surface area proportions of medium starch granules (2.0 μm < D ≤ 8.0 μm) than LN combined with HT exposure (LN-HT) (Table 2), due to the possible improvement of grain filling and somewhat coordinated modification for the contradiction between the proliferation of newly starch granules and the development of extended starch granules under HT exposure.

Our present result regarding the effect of HT exposure on starch ΔH was well in agreement with the previous reports that warming climatic condition (ET) and HT exposure increased starch ΔH and the proportion of larger size starch granules in rice grains (Liu et al., 2017; Jing et al., 2021). However, we did not fully agree with the results of Zhu et al. (2017), who concluded that heavy application of N fertilizer increased Tp and ΔH of milled grains. Our present findings suggested that the effect of panicle N fertilizer on Tp and ΔH also was largely disturbed by environmental temperature during filling period, with the slightly lowering tendency of Tp and ΔH for the increased level of N fertilizer application under NT (Table 4). Recently, Tang et al. (2019) found that the application of nitrogen fertilizer under ET conditions increased BD and decreased setback value of RVA profile, which had the compensative impact for HT-induced deterioration of cooking and eating quality. However, such the compensative effect of N fertilizer on HT-induced deterioration was not clearly shown in our present result based on RVA data (Figure 5 and Table 5), in addition to the deteriorative palatability of cooked rice as reflected by the higher ΔH value and larger average diameter for MN-HT and HN-HT relative to LN-HT (Figures 2, 4 and Table 4). The different viewpoints might be mostly caused by the different warming amplitudes between the ET and the encounter of relatively extreme HT during the grain-filling period.

Rice cultivars with higher grain protein content were mostly showed the inferior palatability than those with the lower and moderate storage protein content (Tan and Corke, 2002), because the higher concentration of grain storage protein inhibited starch digestibility and prevented water absorption in early cooking (Champagne et al., 2009; Lin et al., 2010). Prolamin and glutelin are the two major storage proteins in rice grains (Lin et al., 2010). The previous studies had confirmed that the prolamin in milled rice was hardly digested by humans, and it also increased the hardness of cooked rice (Champagne et al., 2009), while glutelin was favorable for human health, but it deteriorated the appearance of cooked rice (Bao, 2012; Ning et al., 2020). From Table 6, the effects of N fertilizer and temperature on total protein content were basically similar, with the concurrently increased values of glutelin and total protein contents for the rice plants being imposed to HN and/or HT exposure. According to Olsen (2020), the influence of glutelin composition on the pasting property and GT of cereal starch was largely variable, depending on distinct glutelin subunits that possibly interacted with starch granules and water. For achieving the desirable sensory of cooking rice, further studies should be required to unravel the complex linkage between the varying storage protein composition and the palatability of cooking rice as affected by nitrogen, temperature, and their interaction.

## Conclusion

Nitrogen fertilizer, HT exposure, and their interaction had significant effects on milling quality, grain chalky occurrence, starch composition, storage protein content, starch thermal and pasting properties, and the particle size distribution of starch granules. Comparatively, HT exposure had a much more striking impact on starch physicochemical properties than the varying levels of N fertilizer application. The appropriate application amount of N fertilizer partially attenuated the negative effect of HT exposure on milling quality and chalky occurrence, but the application of panicle N fertilizer significantly increased the average particle size of starch granules by the simultaneous increments in the number and surface area proportions of medium starch granules (2.0 μm < D ≤ 8.0 μm). Furthermore, the topdressing of panicle N fertilizer tended to enhance starch gelatinization enthalpy (ΔH) and its setback viscosity in HT-ripening grains. The effects of higher N fertilizer and HT exposure on the contents of grain total protein and glutelin composition were additively increased. Hence, the topdressing of panicle N fertilizer exacerbated the HT-induced deterioration for the cooking and eating quality, rather than alleviating the negative impact of HT exposure on the palatability of cooked rice. On the other hand, the deteriorated property of cooking and eating quality being caused by HT exposure in combination with heavy N fertilizer (HN) was not mainly attributable to the adverse influence of prolamin on palatability of cooking rice.

## Data Availability Statement

The original contributions presented in the study are included in the article/supplementary material, further inquiries can be directed to the corresponding author.

## Author Contributions

YX: data curation and writing—original draft. XG, YZ, and ZW: investigation. ZH: methodology and software. LZ and RJ: methodology. MA: conceptualization and supervision. GP: conceptualization. FC: conceptualization, supervision, and writing, reviewing, and editing. All authors contributed to the article and approved the submitted version.

## Conflict of Interest

The authors declare that the research was conducted in the absence of any commercial or financial relationships that could be construed as a potential conflict of interest.

## Publisher’s Note

All claims expressed in this article are solely those of the authors and do not necessarily represent those of their affiliated organizations, or those of the publisher, the editors and the reviewers. Any product that may be evaluated in this article, or claim that may be made by its manufacturer, is not guaranteed or endorsed by the publisher.
